# Supplementary material for: Limits of Predictability in Commuting Flows in the Absence of Data for Calibration
Source: Sci Rep. 2014 Jul 11;4:5662. doi: 10.1038/srep05662 (PMC4092333; doi:10.1038/srep05662)
Supplement: Supplementary Information — SI Appendix [file srep05662-s1.pdf]

Limits of Predictability in Commuting Flows in the Absence of Data for Calibration

Supplementary Information

Yingxiang Yang, Carlos Herrera, Nathan Eagle, Marta C. Gonzalez

1. Data description and processing

Census data

The LEHD Origin-Destination Employment Statistics (LODES) datasets [4] used by OnTheMap version 6 were reported using 2010 census blocks. Data files are state-based and organized into three types: Origin-Destination (OD), Residence Area Characteristics (RAC), and Workplace Area Characteristics (WAC), all at census block geographic detail. Data is available for most states for the years 2002-2010. The sources of data include:

- Unemployment Insurance (UI) Wage Records reported by employers and maintained by each state.
- The Office of Personnel Management (OPM) provides information on employees and jobs for most Federal employees.
- The Quarterly Census for Employment and Wages (QCEW) provides information on firm structure and establishment location.

What we used in this study is the Origin-Destination (OD) data. The structure of the OD files is in Table S1. We use row one to three in this study.

Bay area cell phone data

The Bay Area cell phone data are collected by a US cell phone operator and contain about half a million customers. Each time a person uses a phone (call/text message/web browsing) the time and the cell phone tower providing the service is recorded. This altogether generates 374 million location records in the three week observational period. A Voronoi tessellation is used to estimate the service area of a cell phone tower. It provides the rough region where a cell phone user can be located by his/her phone usage. Among these half a million users, we select 189,621 most frequent users to study the commuting flows of the Bay Area [16]. For each user, the most frequently connected tower during day time (6am to 6pm) is assigned as the tower of the working location while the most frequently connected tower during night (6 pm to 6 am) is assigned as the home tower location.

Rwanda, Lisbon and Santo Domingo cell phone data

The Rwanda cell phone data are collected by a phone company and contain more than 1 million users. Each time a person calls the time and the cell phone tower providing the service is recorded. There are around 215 million records over a period of 40 days. The entire Rwanda is covered by 196 towers while the capital city Kigali is covered by 47. We select 410,309 most frequent users for this study. The cell phone data from Portugal and Dominican Republic are of similar format. Lisbon has 62,790 frequent users while Santo Domingo has 52,125 frequent users.

2. K-means clustering of blocks

The 2010 Census LEHD Origin-Destination Employment Statistics (LODES) datasets contain home and work location counts at block level. San Francisco has 7,348 blocks while in transportation planning a city is often divided into a much less number of regions [12, 11]. To make the estimation results at different scales comparable, here we adopted k-means clustering [7, 3] to divide the study region into 100 locations. The blocks are clustered according to their geographical locations. The procedure is performed in the following way:

Randomly pick 100 (lon, lat) coordinate pairs in the study region to represent the centers of the clusters. They are denoted as $\mu_k, k = 1, \ldots, 100$. Each block's center location is denoted as a vector $X_i, i = 1, \ldots, number\ of\ blocks$. The goal is to find an assignment of X_i to clusters, as well as a set of vectors $\{\mu_k\}$, such that the sum of the squares of the distances of each data point X_i to its closest vector μ_k, is a minimum. Use 1-of-K coding scheme to represent which cluster each data point X_i should belong to. For each data point X_i, we introduce a corresponding set of binary indicator variables $r_{ik} \in \{0,1\}, k = 1, \ldots, 100$, describing which of the 100 clusters the data point X_i is assigned to. If data point X_i is assigned to cluster k then $r_{ik} = 1$, and $r_{ij=0}\ for\ j \neq k$. The objective function, J, is to minimize the sum of the squares of the distances of each data point to its assigned vector μ_k:

$$J = \sum_{i=1}^{N} \sum_{k=1}^{100} r_{ik} \|X_i - \mu_k\|^2$$

Here the distance measure $\|X_i - \mu_k\|^2$ is the distance of the two coordinate pairs on earth. To find the values for r_{ik} and μ_k, iteratively perform:

1. Keep μ_k fixed, find the r_{ik} values to minimize J. This is simply to find the closest μ_k to each data point X_i.

2. Keep r_{ik} fixed, find the μ_k values to minimize J. J is a quadratic function of μ_k. Take the derivative and with respect to μ_k and set it to zero shows that

$$\mu_k = \frac{\sum_i r_{ik} X_i}{\sum_i r_{ik}}$$

Iteratively perform these two steps until converge.

Fig. S1 (a,b) shows the comparison of San Francisco's blocks before and after clustering.

3. IPF procedure for OD expansion

We use the Bay area as an example to show that cell phone data could provide a good commuting OD seed matrix. In part 1 we have deduced home and work locations for each user. Here a location is a cell phone tower. There are 892 towers in the Bay area while in previous methods we divided San Francisco into 100 locations. In order to match these two different types of divisions we mapped the 892 cell phone towers to the previously defined 100 block clusters to form the 100×100 commuting OD matrix for the cell phone users. We should notice that the

cell phone users we chose are like a sample from the whole population and the sampling rates in different block clusters may differ. In order to get the commuting OD matrix for the whole population from the cell phone user commuting OD matrix, we need to reweight or perform seed matrix expansion on the cell phone user commuting OD matrix. The iterative proportional fitting method is adopted [5].

Iterative proportional fitting is a procedure for adjusting a table of data cells such that they add up to selected totals. Unadjusted data cells may be referred to as "seed", and the selected totals may be referred to as "marginal". In our two dimensional case, the "seed" is the cell phone user commuting OD matrix denoted as t_{ij}, i is the home location while j is the work location. We've shown that population and POI are good representations of trip generation and attraction. We use them to represent the "marginals". The column marginal D_j is the trip attraction of each location and the row marginal O_i is the trip generation of each location. D_j are normalized to have the same sum as O_i. The numerical solution is:

1. $\hat{T}_{ij}^{m} = t_{ij}, m = 0$

2. a1) $For\ i = 1, \dots, N$

    i. Solve for α: $\sum_j \hat{T}_{ij}^{m} \alpha = O_i$

    ii. $\hat{T}_{ij}^{m+1/2} = \hat{T}_{ij}^{m} \alpha$

    a2) $m = m + \frac{1}{2}$

    b1) $For\ j = 1, \dots, N$

    i. Solve for α: $\sum_i \hat{T}_{ij}^{m} \alpha = D_j$

    ii. $\hat{T}_{ij}^{m+1/2} = \hat{T}_{ij}^{m} \alpha$

    b 2) $m = m + \frac{1}{2}$

3. Repeat step 2 until converge.

Some may doubt that the close fit of the expanded Bay Area cell phone user seed matrix to the actual census data is because we used quite accurate marginal (in this case the population density and the density of POIs), so that the seed matrix do not have much influence. We test this assumption by doing the following comparison: compare the travelling distance $P(r)$ distribution of: 1) the census commuting OD data; 2) the cell phone user seed OD matrix without IPF expansion; 3) the IPF expanded cell phone user seed matrix; 4) the IPF expanded random seed matrix. The result is shown in Fig. S2. Among all others, only the IPF expended cell phone user seed matrix gives close fit to the census data. As for the IPF expanded random seed matrix, even though it has accurate marginal, it still deviates from the actual $P(r)$ distribution. In this way the value of both the IPF method and the cell phone user seed matrix are shown.

4. Comparison of the unconstrained with the doubly constrained gravity model

In this section we compare the estimation results of the unconstrained gravity model and the doubly constrained gravity model on cell phone user commuting OD at city level. Here we use the cell phone records because the data is available at different countries so that we can perform a cross culture comparison. We choose 9 cities from the Bay area, Rwanda, Portugal and Dominican Republic: San Francisco, Oakland, San Jose, San Rafael, Lisbon, Kigali, La Romata, Santo Domingo, and Santiago. For each cell phone user we can estimate his/ her home and work location. Aggregating such results gives us the cell phone users' commuting OD matrix. Use the margins (cell phone user commuting trip production and attraction number for each tower) as inputs for the following models.

The unconstrained gravity model takes the form:

$$T_{ij} = \frac{n_i^{\alpha} n_j^{\beta}}{f(r_{ij})}$$

T_{ij} is the flow between location i and j. Each location is a tower. n_i is the number of cell phone users whose home location is tower i, n_j is the number of cell phone users whose working location is tower j. r_{ij} is the distance between them and f is the distance decay function. α and β are parameters to be fitted from data. We adopt the power distance decay function:

$$f(r_{ij}) = r_{ij}^{\gamma}$$

The model turns into:

$$T_{ij} = \frac{n_i^{\alpha} n_j^{\beta}}{r_{ij}^{\gamma}}$$

The parameters α, β, and γ could be estimated using least square linear regression [6] after a simple transformation:

$$\log(T_{ij}) = \alpha \log(n_i) + \beta \log(n_j) - \gamma \log(r_{ij})$$

The inputs of the regression model are T_{ij}, n_i, n_j and r_{ij}, the outputs are estimation results of α, β, and γ.

The α, β, and γ regression results for the 9 cities are in Table S2.

In some other studies [15, 1] a similar regression method is applied. The difference is that trips are divided into short and long trips and the parameters are estimated separately. In [14] the estimations of $[\alpha, \beta, \gamma]$ are $[0.30, 0.64, 3.05]$ for short distances ($r < 119km$) and $[0.24, 0.14, 0.29]$ for long distances.

The doubly constrained gravity model takes the form:

$$T_{ij} = \frac{\alpha_i \beta_j O_i D_j}{r_{ij}^{\gamma}}$$

O_i and D_j are total trip production and attraction volumes at location i and j. For a study region with n locations, there are $2n$ parameters of α_i and β_j, and one parameter of γ. Unlike the unconstrained gravity model, though it has $2n + 1$ parameters, only one parameter γ needs to be predetermined. α_i and β_j can be estimated even without knowing T_{ij} by iterating:

$$\alpha_i = 1/\sum_j \beta_{j\neq i} D_j \, r_{ij}^{\gamma}$$

$$\beta_j = 1/\sum_i \alpha_{i\neq j} O_i \, r_{ij}^{\gamma}$$

Let's use a very simple example to illustrate the algorithm.

Suppose there is an area with 4 zones. Their distance matrix and O_i, D_j are in Table S3.

Initially α_i and β_j are all set to 1 and β_j are updated as:

$$\beta_1 = \frac{1}{1 * 200 * 1.5^2 + 1 * 100 * 2^2 + 1 * 50 * 3.5^2} = 0.00848$$

$$\beta_2 = \frac{1}{1 * 150 * 1.5^2 + 1 * 100 * 2.5^2 + 1 * 50 * 3^2} = 0.01134$$

$$\beta_3 = \frac{1}{1 * 150 * 2^2 + 1 * 200 * 2.5^2 + 1 * 50 * 2^2} = 0.01220$$

$$\beta_4 = \frac{1}{1 * 150 * 3.5^2 + 1 * 200 * 3^2 + 1 * 100 * 2^2} = 0.01681$$

Then α_i are updated:

$$\alpha_1 = \frac{1}{0.01134 * 70 * 1.5^2 + 0.01220 * 250 * 2^2 + 0.01681 * 150 * 3.5^2} = 0.75715$$

$$\alpha_2 = \frac{1}{0.00848 * 30 * 1.5^2 + 0.01220 * 250 * 2.5^2 + 0.01681 * 150 * 3^2} = 1.13500$$

$$\alpha_3 = \frac{1}{0.00848 * 30 * 2^2 + 0.01134 * 70 * 2.5^2 + 0.01681 * 150 * 2^2} = 1.21780$$

$$\alpha_4 = \frac{1}{0.00848 * 30 * 3.5^2 + 0.01134 * 70 * 3^2 + 0.01220 * 250 * 2^2} = 1.14800$$

After 4 iterations α_i and β_j values converge. The final OD matrix is in Table S4.

Here we compare the results from: the unconstrained gravity model with parameters estimated in this study, the unconstrained gravity model with parameters estimated in previous study [14], the

doubly constrained gravity model with parameters estimated in this study. For each model we compare the model estimation results with the cell phone user commuting OD matrix and calculate the correlation between them. Fig. S3 shows how the correlation changes from city to city and from model to model. In all cities the doubly constrained gravity model outperforms the unconstrained gravity model. It has correlation more than 0.8 in all the cities except in Kigali, the capital city of Rwanda. We've mentioned that the commuting flow in Rwanda is special because it's more agglomerated: a few OD pairs have very large flows and these OD pairs are not necessarily close to each other. This makes it hard for gravity model prediction. The comparison of the doubly constrained gravity model and the no gravity model with parameters estimated in this study are in Fig. S4-S5. Again the doubly constrained gravity model prevails at each measurement.

5) A statistical measurement of the commuting distance

As is proposed in some previous studies [2, 8, 9, 10], there may exist some simple scaling for a given region of total area A and population P. The length scale of a region is represented by $\sqrt{A}$. The expected scaling of the total distance travelled by all the population l_d should be of the form:

$$\frac{l_d}{\sqrt{A}} \sim P^{\beta}$$

In one limiting cases, if every individual is going to the nearest neighbor (with a typical distance $\frac{1}{\sqrt{\rho}}$ while $\rho = P/A$ is the average density of the city), $\beta = 1/2$. In another case, if everyone goes randomly, $\beta = 1$. The empirical cases show that the β value is usually around 0.6.

We did the same measurement for the 1000 different regions in the US and the cell phone users in Rwanda, Santo Domingo, and Lisbon. The results are shown in Fig. S5. The corresponding β value is 0.75. Since we are only counting the commuting distance, the larger β value shows that people are willing to travel longer for working than doing other activities.

6) Correlation between supply and demand at different scales

We've used San Francisco, the Bay area, and the west coast of US to show that at large scales population density can represent both commuting trip generation and attraction while at small scales such as within a city the attraction is better represented by distribution of job opportunities, in this case the POI density. Then there remains the question: to which scale can population density represent both trip generation and attraction?

To generalize and quantify this result, we change the scale gradually and sample multiple regions at each scale to observe the change in the correlation of: population – commuting generation, population – commuting attraction, POI – commuting generation, POI – commuting attraction. The result is in Fig. S7. The figure shows clearly that at large scales the four distributions are close to each other, while at smaller scales commuting trip generation is better represented by population density while commuting trip attraction is better represented by POI density.

7) The relation between the number of opportunities and distance

The main difference between the radiation model (also the intervening opportunity model) and the gravity model is the latter measures distance directly while the former represent 'distance' as the number of opportunities in between. Of course we'd expect the number of opportunities and the distance should generally have a positive correlation. But how high this correlation is? Fig. S8 shows the scatter plot of number of opportunities a versus distance r for each OD pair observed in the census in San Francisco, the Bay area, and the west coast. Although the positive correlation is clear, but given a certain distance the range of number of opportunities is large which is caused by the heterogeneity of the density of opportunities.

To calculate the fractal dimension of the POI distribution, we regress a on r by applying $a = \rho r^{d_F}$. ρ is the density of POIs and d_F is fractal dimension. The d_F for the three regions are respectively 1.69, 1.17, 1.60.

8) The form of the λ distribution

In the derivation of the extended radiation model, the chosen $p(\lambda)$ distribution is the exponential distribution, while it is very reasonable to expect people could have either a scale-free or a well defined scale λ value distribution, so we did further explorations on this. In practice, what we cannot observe is each person's actual λ value while what we can observe is the number of opportunities each person has considered before choosing the job destination, which is approximated by the number of point of interests. So the shape of the $P_{>}(a)$ distribution can inform us which λ distribution to use.

If the λ distribution is in a power law form, such as a Pareto distribution:

$$P(\lambda) = \frac{k\lambda_{min}^{k}}{\lambda^{k+1}}$$

Then the probability of not accepting the closest a opportunities is:

$$P_{>}(a) = \int_{\lambda_{min}}^{\infty} e^{-a\lambda} \frac{k\lambda_{min}^{k}}{\lambda^{k+1}} d\lambda$$

$$= -ka^{k}x_{min}^{k}\Gamma[-k, ax]$$

The Γ function is the upper incomplete gamma function, defined as:

$$\Gamma(a,x) = \int_{x}^{\infty} t^{a-1}e^{-t}dt$$

For example, set $k = 3$ and $\lambda_{min} = 1$, the $P_{>}(a)$ plot then becomes the curve shown in Fig. S9.

Comparing with Fig. 3(a) in the main text shows that the shape of this $P_{>}(a)$ distribution is different from the observed $P_{>}(a)$ distribution in the Bay area, West U.S., Portland-Seattle region, and L.A.-Las Vegas region. So we'd expect in regions larger than a city such a Pareto λ

distribution is not suitable. But Fig. S9 shows a similar shape to the observed $P_>(a)$ distribution in San Francisco. As we can observe from Fig. 3(b) in the paper, at the city scale such as San Francisco, it is harder to find a close fit to the empirical data. At this scale the α vs. l relationship shown in Fig. 3(b) is a raw approximation. This might partly be because in such a compact and dense city like San Francisco, an exponential λ distribution is not most suitable, and this leads to an open question that what forms of λ distribution should be used in dense and compact regions or how to better model such cases.

In general, people's λ value distribution should not be changed by how we choose the region scale and zone size. But since in transportation and urban planning, practitioners divide a region into zones; trips within zones or outside the region boundary are not considered. Thus this filters out part of the trips, which causes the λ distribution to change when the region scale and zone granularity change.

On the other hand, people's λ distribution may have a well-defined scale. In this case if we assume that people's λ values are the same, then:

$$P_>(a) = e^{-a\lambda}$$

Again, it differs from the observations in regions larger than a city, but might be suitable for some city scale regions; this is the origin of the introduction of the α parameter to account for the differences of the zones that constitute trip origins and destinations.

To sum up, at city scale we could explore different forms of λ distribution to find out how different characteristics of a city (scale, population density, *etc*) influence the best λ distribution; or to develop data-driven models to justify the form of the resulting distribution within cities.

9) Further comparison between the extended radiation model and reference models

As is shown by Equation (16) in the main text of the paper, in the original radiation model, the flow between two regions i and j is proportional to $\frac{n_j}{a_{ij}^2}$, n_j is the number of opportunities in region j while a_{ij} is the number of opportunities between i and j. The extended radiation model is more flexible in that the flow is proportional to $\frac{n_j}{a_{ij}^k}$, $k \in (1, +\infty)$. This is because:

In the main text equation (16) is:

$$\lim_{\alpha \to 0} \frac{\left(a_{ij} + n_j\right)^\alpha - a_{ij}^\alpha}{a_{ij}^\alpha} = \lim_{\alpha \to 0} \alpha \frac{n_j}{a_{ij}}$$

The derivation process is:

$$\lim_{\alpha \to 0} \frac{\left(a_{ij} + n_j\right)^\alpha - a_{ij}^\alpha}{a_{ij}^\alpha} = \lim_{\alpha \to 0} \left[\left(1 + \frac{n_j}{a_{ij}}\right)^\alpha - 1\right]$$

The Taylor expansion of $\left(1+\frac{n_j}{a_{ij}}\right)^{\alpha}$ is:

$$\left(1+\frac{n_j}{a_{ij}}\right)^{\alpha} = 1+\sum_{n=1}^{\infty}\frac{\alpha(\alpha-1)\dots(\alpha-n+1)}{n!}\left(\frac{n_j}{a_{ij}}\right)^{n}$$

This holds when $\frac{n_j}{a_{ij}} \in (-1,1)$.

α → 0 occurs in dense and compact regions such as San Francisco, in such regions opportunities are more homogeneously distributed, so n_j is at least order smaller than a_{ij}, the first term of the expansion will dominate the value because the expansion term decays as $\left(\frac{n_j}{a_{ij}}\right)^{n}$, so:

$$\left(1+\frac{n_j}{a_{ij}}\right)^{\alpha} = 1+\sum_{n=1}^{\infty}\frac{\alpha(\alpha-1)\dots(\alpha-n+1)}{n!}\left(\frac{n_j}{a_{ij}}\right)^{n} \approx 1+\alpha\frac{n_j}{a_{ij}}$$

Therefore in equation (16):

$$\lim_{\alpha\to 0}\frac{\left(a_{ij}+n_j\right)^{\alpha}-a_{ij}^{\alpha}}{a_{ij}^{\alpha}} = \lim_{\alpha\to 0}\left[\left(1+\frac{n_j}{a_{ij}}\right)^{\alpha}-1\right] \approx \lim_{\alpha\to 0}[1+\alpha\frac{n_j}{a_{ij}}-1] = \lim_{\alpha\to 0}[\alpha\frac{n_j}{a_{ij}}]$$

Take this back to equation (15):

$$P\left(1\middle|n_i,n_j,a_{ij}\right) = \lim_{\alpha\to 0}\frac{[\left(a_{ij}+n_j\right)^{\alpha}-a_{ij}^{\alpha}](n_i^{\alpha}+1)}{\left(a_{ij}^{\alpha}+1\right)[\left(a_{ij}+n_j\right)^{\alpha}+1]} = \lim_{\alpha\to 0}\frac{\left(a_{ij}+n_j\right)^{\alpha}-a_{ij}^{\alpha}}{2}$$

$$= \lim_{\alpha\to 0} a_{ij}^{\alpha}\times\frac{\left(a_{ij}+n_j\right)^{\alpha}-a_{ij}^{\alpha}}{2\times a_{ij}^{\alpha}} = \lim_{\alpha\to 0}\frac{\left(a_{ij}+n_j\right)^{\alpha}-a_{ij}^{\alpha}}{2\times a_{ij}^{\alpha}} = \lim_{\alpha\to 0}[\alpha\frac{n_j}{2\times a_{ij}}]$$

The α value is cancelled out when applying the result of equation (15) to equation (11). In equation (11) the α in the denominator and the numerator will cancel out.

When the parameter $\alpha \to 0$, $k \to 1$. So the minimum value of k is 1, while in some cases (like small and dense cities) the required k value is smaller than 1. This is the reason that we conclude under such scales it is hard to capture the accurate flows with general expressions for the distribution (as opposed to fitting actual trips). More detailed characteristics of the region such as landuse and road networks, as well as a more detailed model, may need to be considered at intra city scales (regions within the daily scale).

The limitations of previous models are further illustrated in Fig. S10. Each black square represents a study region while each blue circle means a populated zone with the same number of population and opportunities. The rest of the region is assumed to be un-populated.

The limitation of the original radiation model is shown by comparing Fig. S10 (a) with Fig. S10 (b). Since the model is not taking distance into account, it will give the same flow estimation

from zone 1 to zone 3 in both sub-figures. In reality because the distance between zone 1 and zone 3 is longer in sub-figure (b), we would expect less people commuting between zone 1 and zone 3.

The limitation of the no constraint gravity model is shown in Fig. S10 (c). Because the distance between zone 1 and zone 3 is the same as the distance between zone 3 and zone 5, the model will give the same flow estimation from zone 1 to zone 3 and from zone 5 to zone 3. But since there is a highly populated region zone 7 between zone 1 and zone 3, we would expect some people originating from zone 1 being attracted to zone 7, so that less people will travel from zone 1 to zone 3. The gravity model cannot handle situations like this.

The extended radiation aims to solve both limitations indicated above. Because the number of opportunities is taken into account directly, it solves the limitation shown in Fig. S10(c). Also the scaling parameter α partly solves the limitation shown in Fig. S10 (a) and (b). In (b) the α value will be larger than the α value in (a), causing more people originating from zone 1 to travel to zone 2 and less people to travel to zone 3.

The above analysis shows that both the borders/interface effect and the distribution of population/opportunities have influences on the applicability of the models. The extended radiation model partly solves the limitations, but there are still some open questions such as:

1. How to better quantitatively measure the homogeneity of the population/opportunity distribution and incorporate a distance function.
2. How to quantitatively measure the influence of the shape of the region.

References

[1] D. Balcan, V. Colizza, B. Gonçalves, H. Hu, J.J. Ramasco, and A. Vespignani. Multiscale mobility networks and the spatial spreading of infectious diseases. *Proceedings of the National Academy of Sciences*, 106(51):21484–21489, 2009.

[2] Marc Barthélemy. Spatial networks. *Physics Reports*, 499(1):1–101, 2011.

[3] Paul S Bradley and Usama M Fayyad. Refining initial points for k-means clustering. In *Proceedings of the Fifteenth International Conference on Machine Learning*, volume 66. San Francisco, CA, USA, 1998.

[4] US Census Bureau. https://explore.data.gov/labor-force-employment-and-earnings/lehd-origin-destination-employment-statistics-lode/zvvq-y3uj/. https://explore.data.gov/Labor-Force-Employment-and-Earnings/LEHD-Origin-Destination-Employment-Statistics-LODE/zvvq-y3uj/, 2012. [Online; accessed 26-Feb-2013].

[5] Stephen E Fienberg and Michael M Meyer. Iterative proportional fitting. Technical report, DTIC Document, 1981.

[6] Robin Flowerdew and Murray Aitkin. A method of fitting the gravity model based on the poission distribution. *Journal of Regional Science*, 22(2):191–202, 1982.

[7] John A Hartigan and Manchek A Wong. Algorithm as 136: A k-means clustering algorithm. *Applied statistics*, pages 100–108, 1979.

[8] Yanqing Hu, Yougui Wang, Daqing Li, Shlomo Havlin, and Zengru Di. Maximizing entropy yields spatial scaling in social networks. *arXiv preprint arXiv:1002.1802*, 2010.

[9] Jean-Paul Hubert and Philippe L Toint. From average travel time budgets to daily travel time distributions: appraisal of two conjectures by koelbl and helbing and some consequences. *Transportation Research Record: Journal of the Transportation Research Board*, 1985(1):135–143, 2006.

[10] Mark L Huson and Arunabha Sen. Broadcast scheduling algorithms for radio networks. In *Military Communications Conference, 1995. MILCOM'95, Conference Record, IEEE*, volume 2, pages 647–651. IEEE, 1995.

[11] Thomas L Magnanti and Richard T Wong. Network design and transportation planning: Models and algorithms. *Transportation Science*, 18(1):1–55, 1984.

[12] Michael D Meyer and Eric J Miller. *Urban transportation planning: A decision-oriented approach*. 2001.

[13] Morton Schneider. Gravity models and trip distribution theory. *Papers in Regional Science*, 5(1):51–56, 1959.

[14] F. Simini, M.C. González, A. Maritan, and A.L. Barabási. A universal model for mobility and migration patterns. *Nature*, 484(7392):96–100, 2012.

[15] Cécile Viboud, Ottar N BjØrnstad, David L Smith, Lone Simonsen, Mark A Miller, and Bryan T Grenfell. Synchrony, waves, and spatial hierarchies in the spread of influenza. *science*, 312(5772):447–451, 2006.

[16] Pu Wang, Timothy Hunter, Alexandre M Bayen, Katja Schechtner, and Marta C González. Understanding road usage patterns in urban areas. *Scientific reports*, 2, 2012.

Tables and Figures

Table S1. Data format description for the OD files

Pos	Variable	Type	Length	
1	w_geocode	Char	15	Workplace Census Block Code
2	h_geocode	Char	15	Residence Census Block Code
3	S000	Num	8	Total number of jobs
4	SA01	Num	8	Number of jobs of workers age 29 or younger
5	SA02	Num	8	Number of jobs for workers age 30 to 54
6	SA03	Num	8	Number of jobs for workers age 55 or older
7	SE01	Num	8	Number of jobs with earnings $1250/month or less
8	SE02	Num	8	Number of jobs with earnings $1251/month to $3333/month
9	SE03	Num	8	Number of jobs with earnings greater than $3333/month
10	SI01	Num	8	Number of jobs in Goods Producing industry sectors
11	SI02	Num	8	Number of jobs in Trade, Transportation, and Utilities industry sectors
12	SI03	Num	8	Number of jobs in All Other Services industry sectors
13	createdate	Char	8	Date on which data was created, formatted as YYYYMMDD

Table S2. Regression parameters for the 9 cities

	San Francisco	Oakland	San Jose	San Rafael	Lisbon	Kigali	La Romata	Santo Domingo	Santiago
α	0.17	0.21	0.16	0.21	0.21	0.16	0.53	0.25	0.34
β	0.09	0.18	0.15	0.23	0.23	0.11	0.33	0.20	0.27
γ	0.46	0.67	0.56	0.73	0.73	0.43	0.91	0.68	0.73

Table S3. Seed sample matrix without expansion

Zone	1	2	3	4	O_i
1	0	1.5	2	3.5	150
2	1.5	0	2.5	3	200
3	2	2.5	0	2	100
4	3.5	3	2	0	50
D_j	30	70	250	150	$Total = 500$

Table S4. Converged sample OD matrix

Zone	1	2	3	4	O_i	α_i
1	0	45	86	19	150	0.71159
2	22	0	120	58	200	1.16540
3	7	20	0	73	100	1.31410
4	1	5	44	0	50	1.07820
D_j	30	70	250	150		
β_j	0.00710	0.01343	0.01291	0.01482		

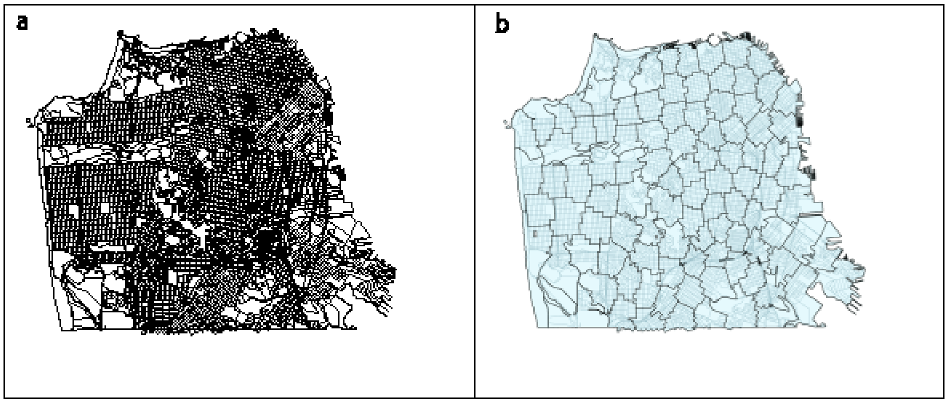

Fig. S1. San Francisco before and after block clustering. (a) The 7,348 blocks of San Francisco. (b) 100 block clusters acquired from k-means clustering

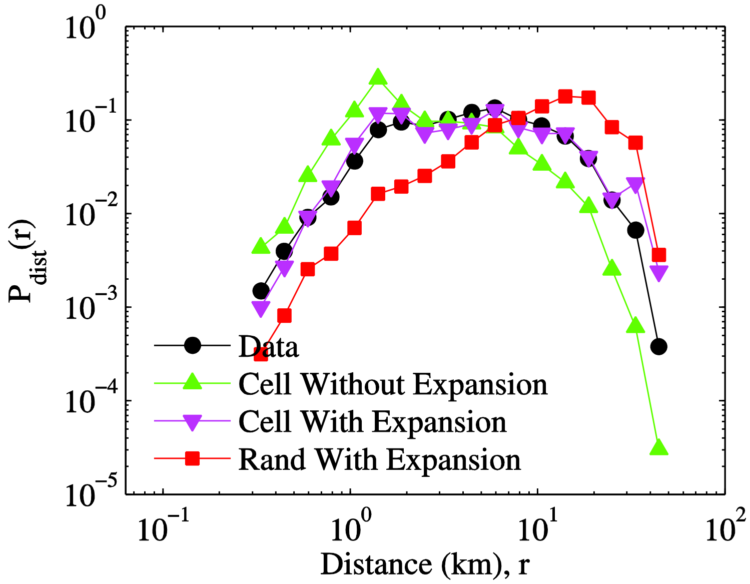

Fig. S2. Comparison of the travelling distance $P(r)$ distributions. The census commuting OD data is in black. The cell phone user seed OD matrix without IPF expansion is in green. The IPF expanded cell phone user seed matrix is in purple. The IPF expanded random seed matrix is in red. Only the IPF expended cell phone user seed matrix gives close fit to the census data. As for the IPF expanded random seed matrix, even though it has accurate marginal, it still deviates from the actual $P(r)$ distribution.

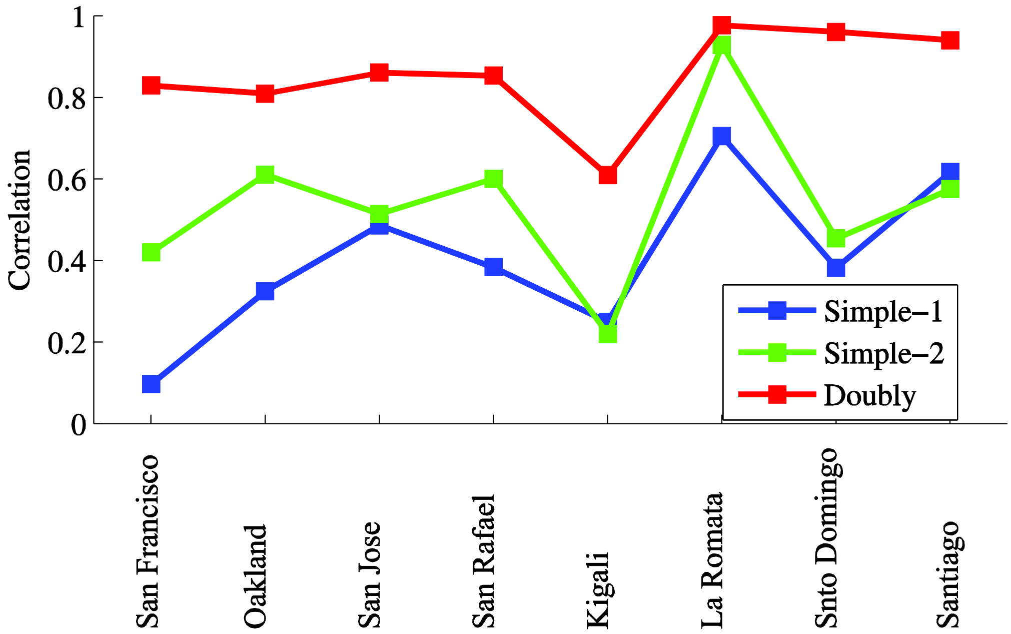

Fig. S3. The correlation between the census commuting OD pair volumes and results from different models. The doubly constrained gravity model's result is in red. The unconstrained gravity model with parameters estimated from a previous study is in blue. The unconstrained gravity model with parameters estimated in this study is in green. In all cities the doubly constrained gravity model outperforms the unconstrained gravity model. It has correlation more than 0.8 with the actual census data in all the cities except Kigali.

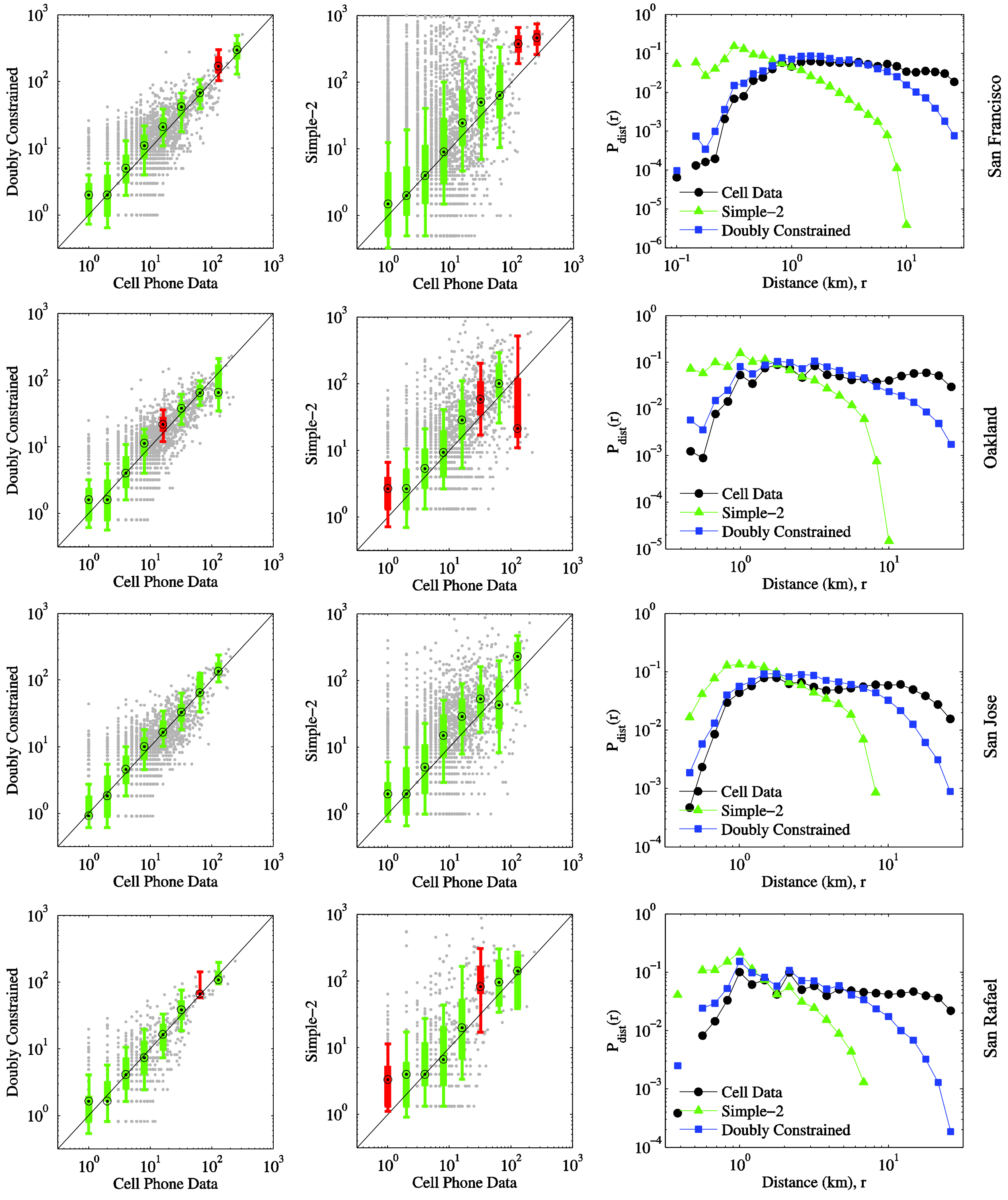

Fig. S4. Further comparison of the two gravity models in US cities: the doubly constrained gravity model and the unconstrained gravity model with parameters estimated in this study. The 4 rows represent the 4 different cities and the three columns show: 1) the comparison between the actual and estimated flow volume from the doubly constrained gravity model; 2) the comparison between the actual and estimated flow volume from the unconstrained gravity model; 3) the travel distance $P(r)$ distribution.

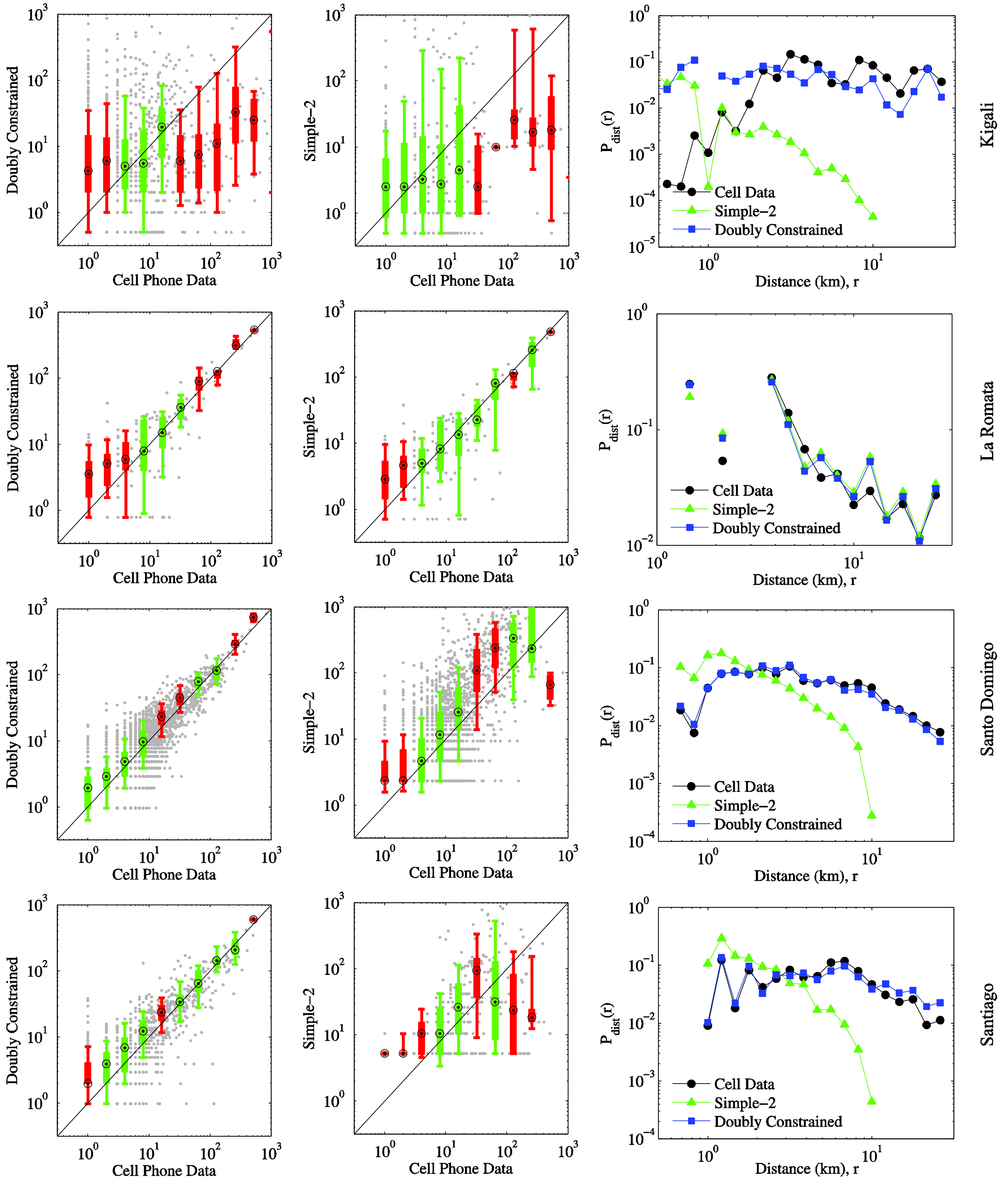

Fig. S5. Further comparison of the two gravity models in other countries: the doubly constrained gravity model and the unconstrained gravity model with parameters estimated in this study. The 4 rows represent the 4 different cities and the three columns show: 1) the comparison between the actual and estimated flow volume from the doubly constrained gravity model; 2) the comparison between the actual and estimated flow volume from the unconstrained gravity model; 3) the travel distance $P(r)$ distribution. Again the doubly constrained gravity model prevails at each measurement.

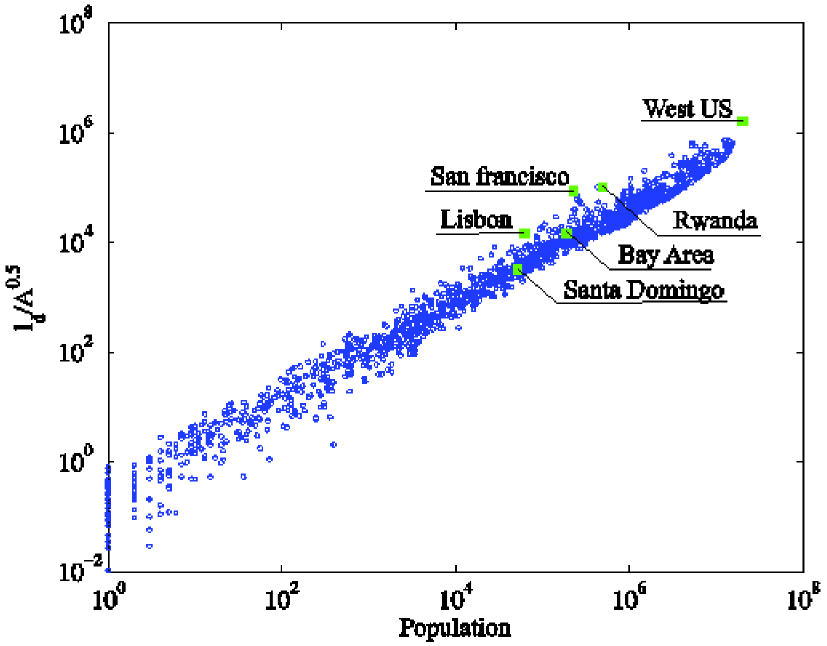

Fig. S6. A scaling measurement of $\frac{l_d}{\sqrt{A}}$ $vs. \beta$. l_d is the total distance travelled by all the population in a region. A is the total area. P is the population. They should follow the scaling relationship: $\frac{l_d}{\sqrt{A}} \sim P^{\beta}$. The blue dots are 1000 randomly selected regions from the US with different sizes and population. Three special cases: the west coast of US, the Bay Area, and San Francisco are marked in green. The measured values for cell phone users in Rwanda, Santo Domingo and Lisbon are also marked. The β value is 0.75, larger than the empirical result of 0.6 which measures travels for all activities, which indicates that people are willing to travel further for commuting than for other activities.

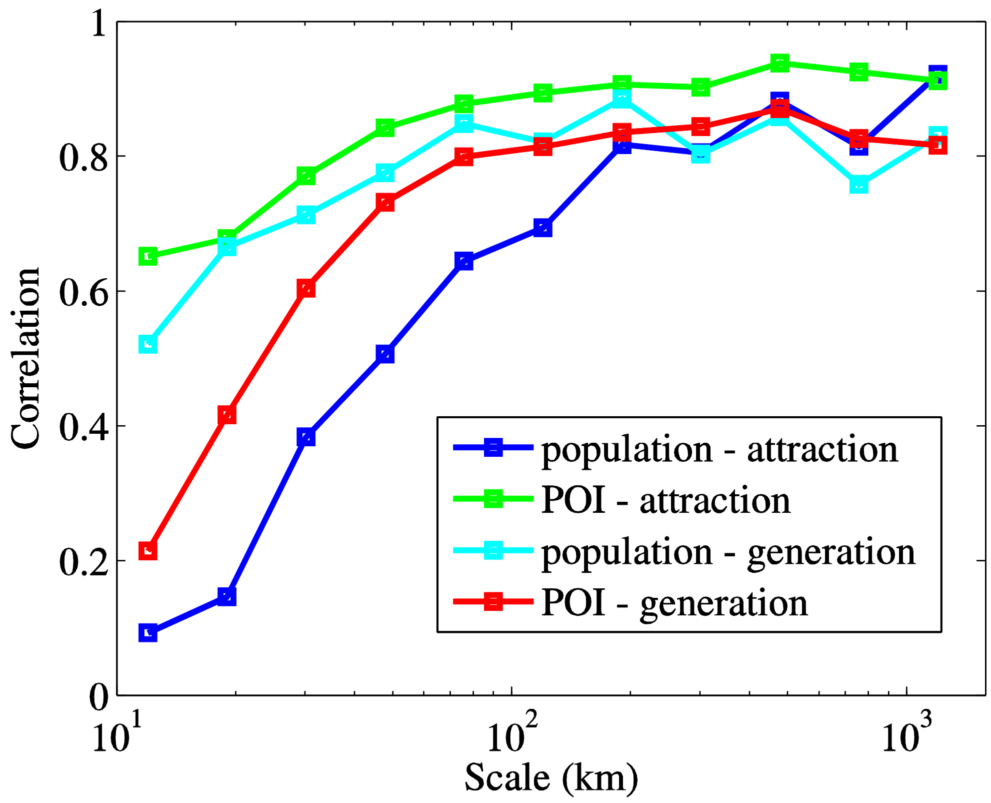

Fig. S7. Correlation between: population – commuting generation, population – commuting attraction, POI – commuting generation, POI – commuting attraction. At small scales commuting trip attraction is better represented by POI density while at large scales these four distributions have high correlation between each other.

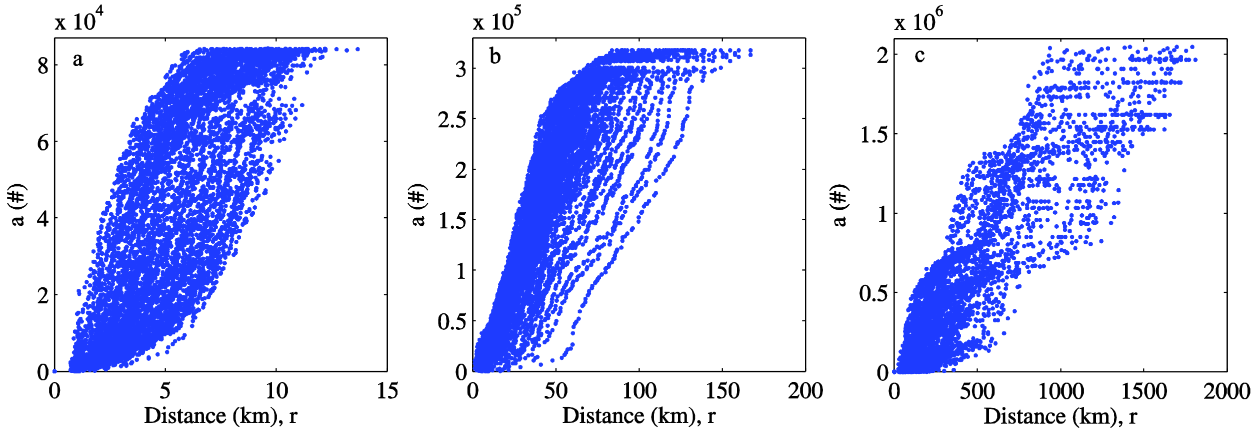

Fig. S8. Scatter plot of the number of opportunities a versus distance r for San Francisco, the Bay area, and the west coast of US.

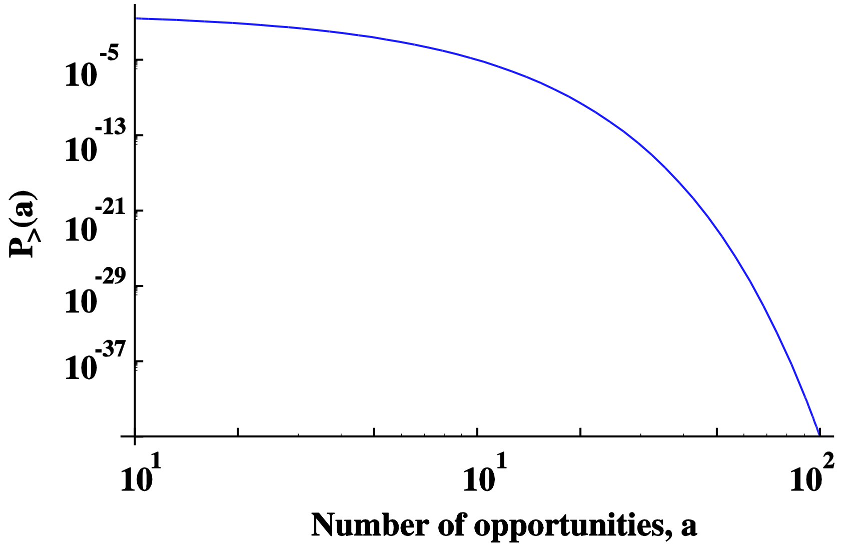

Fig. S9. $P_>(a)$ distribution when λ obeys a Pareto distribution

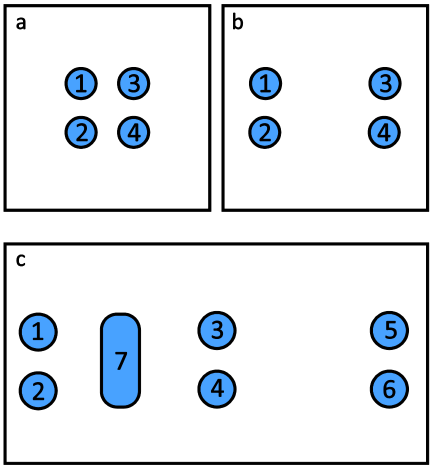

Fig. S10. Illustration of the limitations of the reference models. Each black square represents a study region while each blue circle means a populated zone with the same number of population and opportunities. The rest of the region is assumed to be un-populated.
